# Effect of functional food ingredients on gut microbiota in a rodent diabetes model

**DOI:** 10.1186/s12986-020-00496-2

**Published:** 2020-09-18

**Authors:** Ingrid S. Surono, Ata Aditya Wardana, Priyo Waspodo, Budi Saksono, Jessica Verhoeven, Koen Venema

**Affiliations:** 1 Food Technology Department, Faculty of Engineering, Bina Nusantara University, 11480 Jakarta, Indonesia; 2 Research Center for Biotechnology, Lembaga Ilmu Pengetahuan Indonesia, Jalan Raya Bogor Km 46, Cibinong, 16911 Indonesia; 3 Centre for Healthy Eating & Food Innovation, Maastricht University - Campus Venlo, Venlo, The Netherlands

**Keywords:** Taro-starch, Prebiotic, Gut microbiota, Diabetes model, Functional food, Obesity

## Abstract

**Background:**

The gut microbiota has been shown to be involved in the development and severity of type 2 diabetes. The aim of the present study was to test the effect of 4-week functional food ingredient feeding, alone or in combination, on the gut microbiota composition in diabetic rats.

**Methods:**

Streptozotocin (STZ)-induced diabetic rats were treated for 4 weeks with (1) native taro starch, (2) modified taro-starch, (3) beet juice, (4) psicose, (5) the probiotic *L. plantarum* IS-10506, (6) native starch combined with beet juice, (7) native starch to which beet juice was adsorbed, (8) modified starch combined with beet juice or (9) modified starch to which beet juice was adsorbed, to modulate the composition of the gut microbiota. This composition was evaluated by sequencing the PCR amplified V3–V4 region of the 16S rRNA gene.

**Results:**

The next-generation sequencing showed beneficial effects particularly of taro-starch feeding. Operational taxonomic units (OTUs) related to health (e.g. correlating with low BMI, OTUs producing butyrate) were increased in relative abundance, while OTUs generally correlated with disease (e.g. Proteobacteria) were decreased by feeding taro-starch.

**Conclusion:**

The results of study show that a 4-week intervention with functional food ingredients, particularly taro-derived starch, leads to a more healthy gut microbiota in rats that were induced to be diabetic by induction with STZ.

## Background

Type 2 diabetes (T2D) increases at a dramatic pace worldwide. The global incidence of T2D is predicted to reach 360 million cases by the year 2030 [1], on a projected world population of 8.5 billion [2]. Although it is commonly accepted that an increase in energy intake and a decrease in energy expenditure are the leading causes of obesity and associated with that T2D, metabolic syndrome and cardiovascular disease, recently the gut microbiota has been shown to play a role as well [3–6]. The gut microbiota composition and/or activity can be changed using functional food ingredients. The vital role of food for prevention and treatment of T2D needs proper attention, such as in the development of dietary components that positively influence postprandial glycaemia and through this their potential to reduce the impact of T2D. On top of that, effects through the modulation of the gut microbiota need to be considered.

Indonesia is rich in biodiversity, including a variety of local tubers, which are currently underutilized, despite their potential functional properties, and widespread use in the past. One of these interesting tubers is Cocoyam or taro, which belongs to the monocotyledonous family Araceae (the aroids). Taro used to be an important ethnic root crop throughout Asia. It was cultivated and used as a staple crop in various parts of the humid tropics and sub-tropics, as it adapts well to different agro-climatic conditions [7–9]. Its utilization is also related to the culture of a region, hence, taro is very important for community-life [10]. Keirke et al. [11] reported that Indonesia has the highest taro diversity in the world, and can be found in areas in Borneo, Java, Sumatra, and Sulawesi [12]. Taro traditionally was used as an alternative carbohydrate source to reduce dependence on rice. We have shown recently that taro contains resistant starch (RS) (manuscript submitted for publication), which reaches the colon and can contribute to the modulation of the gut microbiota.

Recently, in an attempt to produce functional foods aimed at low calorie and less sugar intake for T2D, D-psicose, a rare monosaccharide also known as D-allulose, is considered as a substitute for sugar with proven antihyperglycemic, antihyperlipidemic, and anti-inflammatory effects [13, 14].

Probiotics are defined as “life microorganisms, which when administered in adequate amounts, have a beneficial effect on the host” [15]. Probiotic strains have been isolated from dadih, a traditional fermented buffalo milk, produced in West Sumatra [16]. Dadih has been shown to reduce adiposity, weight gain and adiposity inflammation in high fat induced obese rats [17]. The microbes present in dadih, amongst which *L. plantarum* IS-10506 [16, 18, 19], may contribute to this beneficial effect.

Beet juice is rich in including phenolic acids, flavonoids and betalains [20], and beet juice has a high total antioxidant capacity and total polyphenol content [21]. Polyphenols including flavonoids, phenolic acids, proanthocyanidins and tannins have been suggested to be able to modify postprandial (hyper)glycaemia [22, 23] by inhibiting carbohydrate digestion, reducing glucose absorption in the intestines, stimulation of insulin release from pancreatic β-cells, modulation of hepatic glucose output, activation of insulin receptors, and/or modulation of glucose uptake in insulin-sensitive cells [24, 25]. Polyphenols are not very well absorbed in the small intestine, and reach the colon where the can modulate the gut microbiota. Therefore, beet juice is an interesting food model to investigate any influence of its bioactive components on the glycaemic response, either by direct inhibition of glucose uptake or by indirect action affecting insulin sensitivity, whether or not through modulation of the gut microbiota.

In a previous study (submitted for publication), we reported the effect of (modified) taro starch, psicose, the probiotic *L. plantarum* IS-10506 and phytonutrients of beet juice, alone or in combination on the gut microbiota composition in streptozotocin-induced T2D rats, in a pilot-study of 1 week of feeding. The aim of the current study is to extend the data with results after 4 weeks of feeding these functional food ingredients.

## Materials and methods

### Materials

Streptozotocin (STZ; catalogue number ALX-380-010-G001) was purchased from Enzo Life Science (NY, USA). Psicose was from Merck (Darmstadt, Germany; catalogue number P8043). *L. plantarum* IS-10506 was cultured and prepared as described before [16].

### In vivo animal trial

#### Animals and housing

Because in Indonesia only the Ministry of Health and universities with a medical faculty have ethical committees (and this excludes the universities participating in this study), the ethical committee of the Faculty of Medicine of the University of Indonesia (the most reputable committee) was chosen to assess the animal trial. All animal care procedures were conducted under the animal protocol approved by the ethical committee (Ref: 1196/UN2.F1/ETIK/2018) under approval number 18-09-1045. Male Sprague Dawley rats were purchased from the Animal Experimental Laboratory National Agency of Drug and Food Control (Jakarta, Indonesia) at 6 weeks of age and were allowed to adapt for 14 days. They were housed in individual cages and maintained with a 12-h light/dark cycle, at 21–23 °C and 55% ± 5% humidity. All rodents were given ad libitum access to water and commercially available rat normal pellet diet (NPD) purchased from local market, during a 14 days acclimatization period, prior to the dietary manipulation. Subsequently, purified Rodent Diet AIN-93 M, a modified AIN-76A standard diet (American Institute of Nutrition) [26] was provided to the rats as a control.

#### Diets

Native taro starch “HASIL BUMIKU” was purchased from a local supplier (Kusuka Ubiku) in Bantul, Yogyakarta, Central Java, Indonesia. Modified taro-starch was manufactured by the autoclave-cooling method according to a modification of the method of Zhao and Lin [27]. In brief, taro starch was blended with distilled water based on the ratio 1:3.5, and the blend was then gelatinized using pressure-heated instrument at 121 °C for 30 min and cooled to 4 °C, with a repetition of two cycles. Afterwards, the retrogradaded starch was dried using a fan-assisted oven at 60 °C for 16 h after which it was allowed to cool at room temperature for 24 h, and subsequently grinded, and sieved using a 60 mesh.

 Beet juice was adsorbed to both native and modified taro starch by absorbing beetroot juice, at a ratio of 1:1, and then drying in an oven at 40 °C for 16 h. These were prepared and fed according to the dose of beet juice of 6 ml/day, but in adsorbed beet juice form.

Beet juice (6 ml/day) was also provided separately, as well as in combination with native and modified starch (non-adsorbed). The probiotic *L. plantarum* IS-10506 was given by gavage at 10^10^ colony forming units/day.

Rats were first provided modified AIN-93M by replacing corn starch with the respective taro starch, and replacing sucrose and cellulose with maltodextrin. This ration was provided for 3 days, with 25% incremental increasing dose of the modified AIN (at 75, 50 and 25% commercial rat pellet diet and 25, 50, and 75% dietary intervention formulation for the 3 days, respectively). Beet juice and probiotic were added to AIN-93M diet where applicable by gavage.

#### Development of type 2 diabetes by STZ-treatment

Four rats in each group were allocated to the dietary treatments. Then the rats were injected intraperitoneally (i.p.) with 120 mg kg^−1^ nicotinamide in 0.9% NaCl, followed after 15 min by STZ in citrate buffer pH 4.4 (70 mg kg^−1^), and four days after the STZ injection, fasting and postprandial blood glucose levels were measured using a Freestyle glucose meter (Easy Touch GCU 3 in 1) from a puncture at the tip of the tail. The rats with a fasting glucose of ≥ 100 mg dl^−1^ and/or postprandial blood glucose levels of ≥ 140 mg dl^−1^ were considered as type 2 diabetic, and those rats which had not yet developed T2D within these 4 days were injected for the second time with 120 mg kg^−1^ nicotinamide and STZ in citrate buffer pH 4.4 (70 mg kg^−1^).

After confirmation of T2D, the 8 week old rats of 190–220 g were divided into 10 groups of n = 4 each, namely:

1. AIN-93 M (control),
2. AIN-93 M with two times in a day 3 ml psicose by gavage (psicose),
3. AIN-93 M with two times in a day 3 ml beetroot juice by gavage (beet juice),
4. native taro starch (native starch),
5. modified taro starch (modified starch),
6. native taro starch with beetroot juice adsorbed (native beet adsorbed),
7. modified taro starch with beetroot juice adsorbed (modified beet adsorbed),
8. native taro starch combined with two times in a day 3 ml beetroot juice by gavage (native + beet),
9. modified taro starch combined with two times in a day 3 ml beetroot juice by gavage (modified + beet), and
10. *L. plantarum* IS-10506 (probiotic).

The rats were allowed to continue to feed on their respective diets until the end of the study. Food and water intake were monitored every day. Bodyweight was monitored weekly. The average food intake per rat was calculated. Fecal pellets were collected at the end of 1 week of feeding, as well as after 4 weeks.

### Extraction of nucleic acids

DNA extraction of feces samples was performed using the Quick-DNA™ Fecal/Soil Microbe Miniprep Kit (Zymo Research) according to manufacturer’s instructions, using the Precellys 24 tissue homogenizer (Bertin Instruments, Montigny-le-Bretonneux, France), applying 3 cycles of 30 s each, with 5 min cooling on ice in between.

### PCR-amplifying the V3-V4 region of the 16S rRNA gene and next generation sequencing

Illumina 16S rRNA gene amplicon libraries were generated and sequenced at BaseClear (Leiden, the Netherlands). In short, barcoded amplicons from the V3-V4 region of 16S rRNA genes were generated using a 2-step PCR. 10–25 ng isolated genomic DNA was used as template for the first PCR with a total volume of 50 μl using the 341F (5′-CCTACGGGNGGCWGCAG-3′) and the 785R (5′-GACTACHVGGGTATCTAATCC-3′) primers appended with Illumina adaptor sequences (Illumina, San Diego, CA, USA). PCR products were purified and the size of the PCR products were checked on Fragment analyzer (Advanced Analytical Technologies, Heidelberg, Germany) and quantified by fluorometric analysis. Purified PCR products were used for the 2nd PCR in combination with sample-specific barcoded primers (Nextera XT index kit, Illumina). Subsequently, PCR products were purified, checked on a Fragment analyzer (Advanced Analytical Technologies) and quantified, followed by multiplexing, clustering, and sequencing on an Illumina MiSeq with the paired-end (2x) 300 bp protocol and indexing.

### Sequence processing and analyses

The sequencing run was analyzed with the Illumina CASAVA pipeline (v1.8.3) with demultiplexing based on sample-specific barcodes. The raw sequencing data produced was processed removing the sequence reads of too low quality (only "passing filter" reads were selected) and discarding reads containing adaptor sequences or PhiX control with an in-house filtering protocol. A quality assessment on the remaining reads was performed using the FASTQC quality control tool version 0.10.0. Subsequently, the sequences were further analyzed using the Quantitative Insights Into Microbial Ecology (QIIME) software pipeline, version 1.9.1 [28] for α- and β-diversity and (un)weighted principal coordinate analysis. The software package R (3.5.0) (R Core Team 2013) was used to determine correlations between OTUs and treatments. Statistical analyses were performed with RStudio (1.0.153). Kruskal–Wallis correlations were calculated between OTUs and non-continuous values (treatments). Multiple comparison was corrected using the false discovery rate (FDR), and *q* values (adjusted *p* values) were considered significantly different at *q* < 0.05.

### Data availability

The datasets are available from the corresponding author on reasonable request. Raw sequences have been deposited in the European Nucleotide Archive under submission number PRJEB39722: (https://www.ebi.ac.uk/ena/browser/view/PRJEB39722).

## Results and discussion

**Fig. 1:**
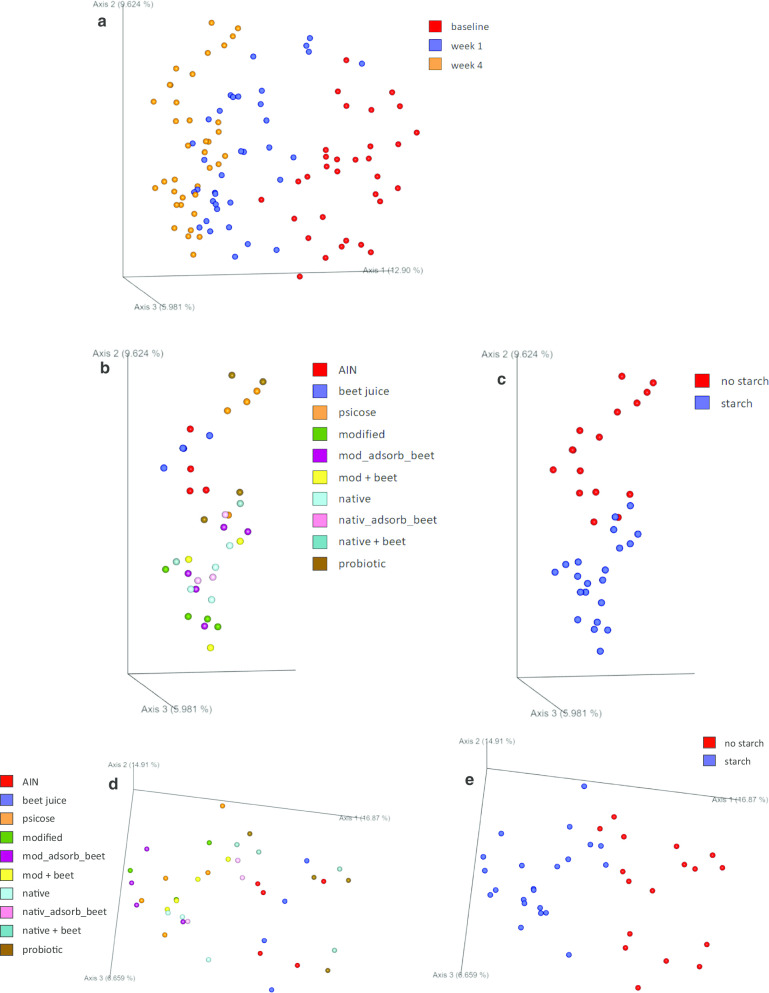
Principal coordinate analysis of unweigthed UniFrac of **a** baseline, week 1 and week 4 samples; **b** the same plot, but with baseline and week 1 samples removed, clustered according to treatment; **c** same as **b** but then clustered according to whether rats were fed starch in their diet or not. Focusing on the week 4 data graphs **d**, **e** the unweighted UniFrac clustered according to treatment and whether rats were fed starch in their diet or not, respectively

STZ induced diabetes in the rats, as indicated by the average fasting blood glucose concentrations of 149 ± 6.3 mg/dl. At this point, rats were randomly divided over the 10 groups, and fed their respective diet for 4 weeks. After 4 weeks of treatment with the different diets, the microbiota composition was clearly different than that at baseline (Fig. 1a). The principal coordinate analysis (PCoA) plot of the unweighted UniFrac β-diversity analysis (Fig. 1a) shows that even after 1 week there are already differences in the gut microbiota, as reported by us before (manuscript submitted). We will focus here on the week 4 data. The same PCoA plot, but then with the baseline and week 1 data removed, shows a separation at week 4 by treatment (Fig. 1b), but especially when the treatments are clustered by containing starch or not (Fig. 1c). This is also the case if only the samples of week 4 are plotted in the unweighted UniFrac β-diversity analysis (Fig. 1d, e for treatment and starch or not, respectively).

**Fig. 2:**
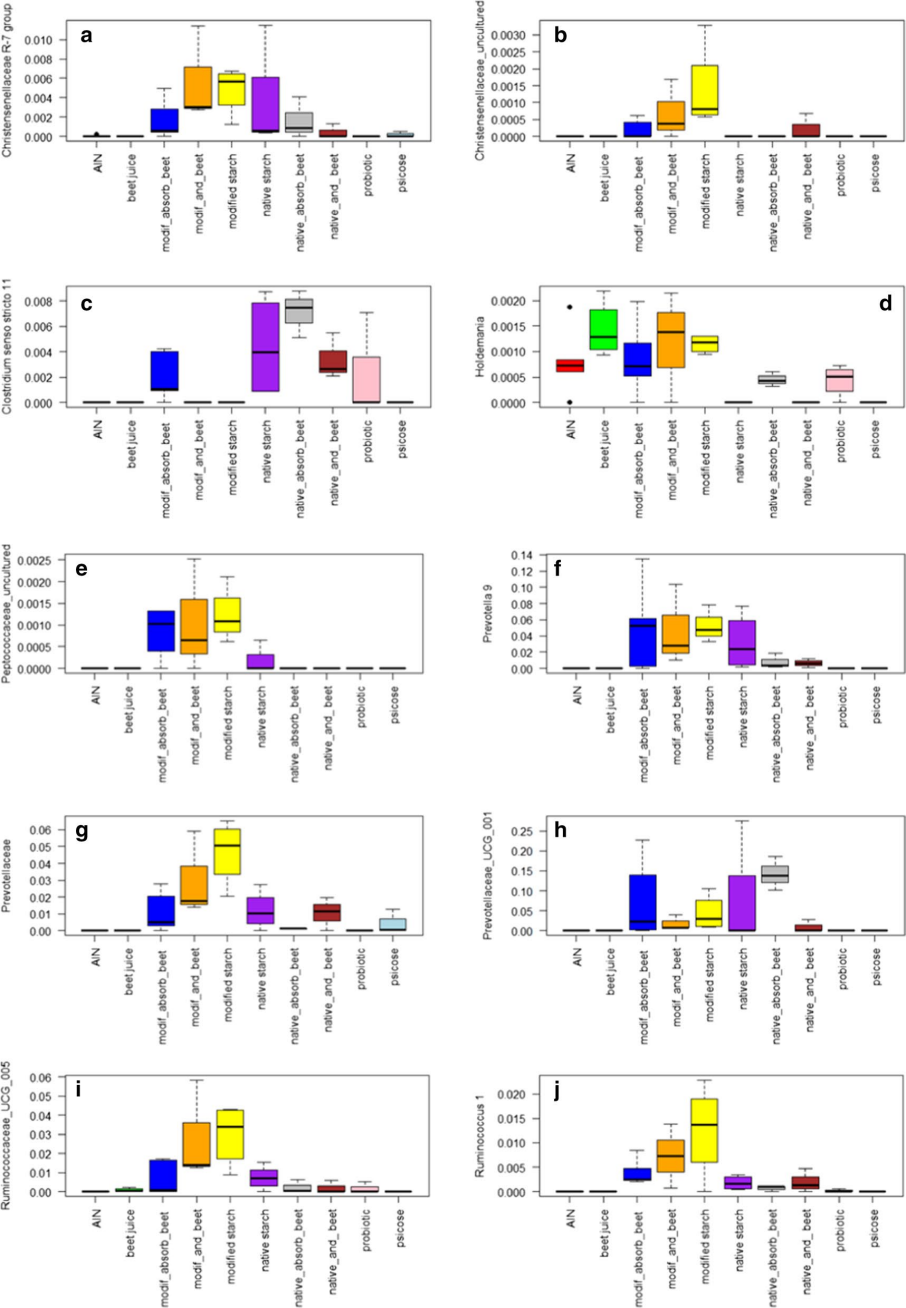
Box-plot representation of OTUs that were significantly different (*q* < 0.05; Table 1A) at the genus of family level after Kruskal–Wallis analysis when compared for dietary treatment. **a** *Christensenellaceae* R-7 group; **b** uncultured species of the *Christensenellaceae* family; **c** *Clostridium *sensu stricto 11; **d** *Holdemania*; **e** uncultured species of the *Peptococcaceae* family; **f** Prevotella 9; **g** unknown species of the *Prevotellaceae* family; **h** *Prevotellaceae* UCG-001; **i** *Ruminococcaceae* UCG-005; **j** *Ruminococcus* 1

**Fig. 3:**
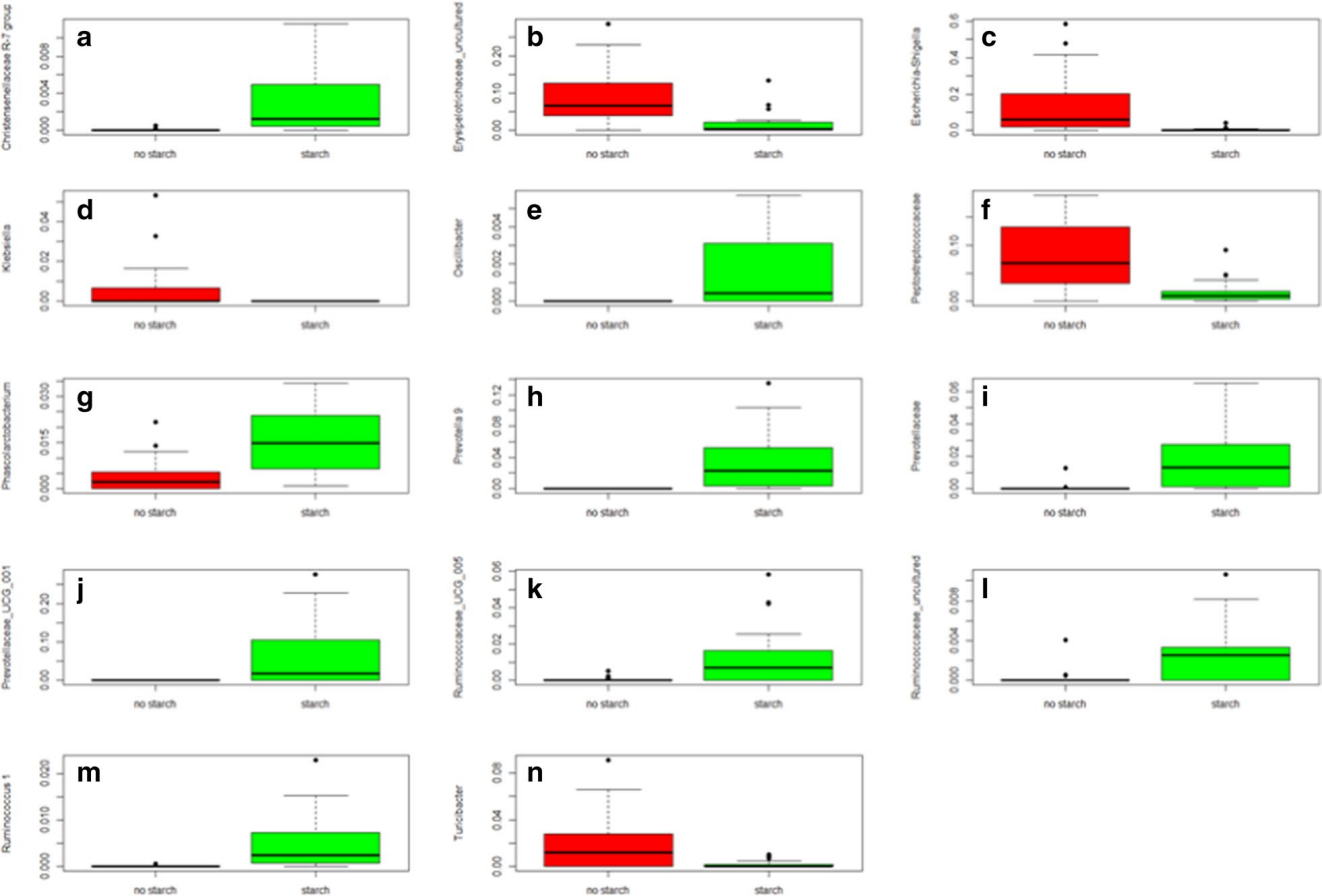
Box-plot representation of OTUs that were significantly different (*q* < 0.05; Table 1B) at the genus or family level after Kruskal–Wallis analysis when compared whether rats were fed starch in their diet or not. **a** *Christensenellaceae* R-7 group; **b** uncultured species of the *Erysipelotrichaceae* family; **c** *Escherichia-Shigella*; **d** *Klebsiella*; **e** *Oscillibacter*; **f** unknown species of the *Peptostreptococcaceae* family; **g** *Phascolarctobacterium*; **h** *Prevotella* 9; **i** unknown species of the *Prevotellaceae* family; **j** *Prevotellaceae* UCG-001; **k** *Ruminococcaceae* UCG-005; **l** uncultured species of the *Ruminococcaceae* family; **m** *Ruminococcus* 1; **n** *Turicibacter*

Kruskal–Wallis analysis revealed the significant OTUs (corrected for multiple comparisons; *q* value < 0.05) that were different between treatment (Fig. 2; Table 1A) or whether rats were fed starch or not (Fig. 3; Table 1B). Six of the OTUs that were significantly different when tested against treatment (Table 1A), were also different when tested for starch, indicating that starch treatment drove the separation between the samples for these 6 OTUs. The relative abundance (RA) of these 6 OTUs, *Christensenellaceae* R-7 group, Prevotella 9, an unknown species of the *Prevotellaceae* family, *Prevotellaceae* UCG-001, and *Ruminococcaceae* UCG-005; and *Ruminococcus* 1, were all increased after starch feeding (Fig. 3a, h–k, m, respectively). However, in most cases the stimulation was higher after modified starch feeding (irrespective of the additional feeding or adsorption of beet juice) than after native starch feeding (Fig. 2a, f–j, respectively). Starch also increased the RA of *Oscillibacter*, *Phascolarctobacterium*, and an uncultured species of the *Ruminococcaceae* family (Fig. 3e, g, l, respectively). These were not significantly different when tested between treatments (although the *p* values were < 0.05 for *Phascolarctobacterium* and the *Ruminococcaceae* species, after multiple correction the *q* value was not). *Christensenellaceae* has been shown to be enriched in individuals with low body mass index [29]. Members of the *Ruminococcaceae* family (including *Oscillibacter*) have been well known to be involved in the production of butyrate, an important health promoting microbial metabolite [30] produced by the gut microbiota, particularly known to be produced upon starch feeding [31, 32]. Members of *Prevotellaceae* are commonly found in the digestive tracts of people who maintain a diet low in animal fats and high in carbohydrates [33]. *Phascolarctobacterium* has been associated with low C-reactive protein in premenopausal women [34].

**Table 1:** OTUs that were significantly different (*q* < 0.05) after Kruskal–Wallis analysis, with corresponding *p* values and *q* values, when compared for treatment (A) or whether rats were fed starch or not (B)

OTU	*p *value	*q *value
(A)		
Bacteroidetes;….;D_4__Prevotellaceae;D_5__Prevotella 9	0.0004	0.024
Firmicutes;….;D_4__Clostridiaceae 1;D_5__Clostridium sensu stricto 11	0.0005	0.024
Bacteroidetes;….;D_4__Prevotellaceae;D_5__Prevotellaceae UCG-001	0.0008	0.025
Bacteroidetes;….;D_4__Prevotellaceae;__	0.0014	0.027
Firmicutes;….;D_4__Peptococcaceae;D_5__uncultured	0.0013	0.027
Firmicutes;….;D_4__Christensenellaceae;D_5__Christensenellaceae R-7 group	0.0021	0.029
Firmicutes;….;D_4__Ruminococcaceae;D_5__Ruminococcus 1	0.0018	0.029
Firmicutes;….;D_4__Christensenellaceae;D_5__uncultured	0.0028	0.034
Firmicutes;….;D_4__Ruminococcaceae;D_5__Ruminococcaceae UCG-005	0.0045	0.045
Firmicutes;….;D_4__Erysipelotrichaceae;D_5__Holdemania	0.0041	0.045
(B)		
Bacteroidetes;…;D_4__Prevotellaceae;D_5__Prevotella 9	1.0E−07	1.0E−05
Bacteroidetes;…;D_4__Prevotellaceae;D_5__Prevotellaceae UCG-001	2.4E−06	7.9E−05
Firmicutes;…;D_4__Ruminococcaceae;D_5__Ruminococcus 1	2.0E−06	7.9E−05
Bacteroidetes;…;D_4__Prevotellaceae;__unknown	8.3E−06	0.0002
Firmicutes;…;D_4__Christensenellaceae;D_5__Christensenellaceae R-7 group	1.1E−05	0.0002
Proteobacteria;…;D_4__Enterobacteriaceae;D_5__Escherichia-Shigella	1.6E−05	0.0003
Firmicutes;…;D_4__Peptostreptococcaceae;__unknown	8.1E−05	0.0011
Firmicutes;…;D_4__Ruminococcaceae;D_5__Ruminococcaceae UCG-005	0.0002	0.0028
Firmicutes;…;D_4__Ruminococcaceae;D_5__Oscillibacter	0.0004	0.0037
Firmicutes;…;D_4__Erysipelotrichaceae;D_5__uncultured	0.0004	0.0037
Proteobacteria;…;D_4__Enterobacteriaceae;D_5__Klebsiella	0.0004	0.0037
Firmicutes;…;D_4__Ruminococcaceae;D_5__uncultured	0.0005	0.0038
Firmicutes;…;D_4__Erysipelotrichaceae;D_5__Turicibacter	0.0005	0.0038
Firmicutes;…;D_4__Acidaminococcaceae;D_5__Phascolarctobacterium	0.0005	0.0038

Starch feeding led to a reduction in RA of an uncultured species of the *Erysipelotrichaceae* family, *Escherichia-Shigella*, *Klebsiella*, an unknown species of the *Peptostreptococcaceae* family, and *Turicibacter*. Members of the family *Erysipelotrichaceae* (incl. Turicibacter) have been shown to be increased in the mouse gut microbiota for mice switched to diets high in fat, and appear to be highly immunogenic and can potentially flourish post-treatment with broad spectrum antibiotics [35, 36]. Therefore, a reduction in these OTUs is interpreted to be beneficial. Similarly, the reduction of the RA of the Proteobacteria *Klebsiella* and *Escherichia-Shigella* is considered to be beneficial. Moreover, a reduction in *Peptostreptococcaceae*, which has been shown to be over-represented in the guts of colorectal cancer patients [37], appears to be beneficial too.

Overall, the changes in relative abundances discussed above induced by taro-starch feeding seem to be beneficial for health, although currently most of the benefits described have been through associations and not cause-and-effect relationships yet.

In addition to the OTUs that were significantly different in abundance upon starch feeding, Fig. 2 list 4 more OTUs (in addition to the 6 that were also significant when testing for starch feeding) that are significantly different when comparing overall treatments. One of these, an uncultured species of the *Christensenellaceae* family (Fig. 2b) seemed to be mostly stimulated by starch, but primarily by modified taro-starch, which may be why it is not significant when comparing for starch feeding versus not. The other 3 are *Clostridium *sensu stricto 11, *Holdemania* and an uncultured species of the *Peptococcaceae*. *Clostridium *sensu stricto 11 (Fig. 2c) is primarily stimulated by native starch (in the presence and absence of beet juice), but also by modified starch with beet juice adsorbed, as well as by feeding *L. plantarum* IS-10506. For *Holdemania* no clear health effects have been described. It is reduced (compared to AIN) in native starch and native starch combined with beet juice, and when psicose is fed (Fig. 2d). Members of the family *Peptococcaceae* have been associated with stress in pregnant women: higher RA correlated with reduced prenatal stress [38]. The RA of the uncultured OTU was primarily increased by modified taro-starch.

### Conclusion

In conclusion, particularly taro-starch feeding led to changes in the gut microbiota of the rats with induced diabetes that could be related to beneficial changes to health.

## Data Availability

The datasets used and/or analysed during the current study are available from the corresponding author on reasonable request.

## Abbreviations

AIN: American Institute of Nutrition
BMI: Body mass index
FDR: False discovery rate
OTU: Operational taxonomic unit
PCoA: Principal coordinate analysis
PCR: Polymerase chain reaction
QIIME: Quantitative insights into microbial ecology
RA: Relative abundance
RS: Resistant starch
STZ: Streptozotocin
T2D: Type 2 diabetes
